# Tolerability on Serious Adverse Events of First-Line Bevacizumab and Cetuximab for RAS Wild-Type Metastatic Colorectal Cancer: A Systematic Review and Meta-Analysis

**DOI:** 10.3390/healthcare10020217

**Published:** 2022-01-23

**Authors:** Yu Na Han, Yeo Jin Choi, Sandy Jeong Rhie

**Affiliations:** 1 Graduate School of Converging Clinical & Public Health, Ewha Womans University, Seoul 03760, Korea; yuna2234@hanmail.net; 2 Graduate School of Clinical Pharmacy, CHA University, Seongnam 13488, Korea; yjchoi@cha.ac.kr; 3 College of Pharmacy, Graduated School of Pharmaceutical Sciences, Ewha Womans University, Seoul 03760, Korea

**Keywords:** adverse events, bevacizumab, cetuximab, metastatic colorectal cancer, medication safety

## Abstract

Proper medication management is crucial in metastatic colorectal cancer because of its substantially low survival rate. There has been advancing evidence on the efficacy of the two most prescribed targeted agents (bevacizumab and cetuximab); however, comprehensive analyses on their safety are limited. This study aims to comprehensively assess the clinical safety of first-line bevacizumab and cetuximab-based chemotherapy in unresectable RAS wild-type metastatic colorectal cancer patients and to provide guidance on the selection of appropriate targeted therapeutic agents. Keyword searches of MEDLINE, Cochrane Library, and ClinicalKey were conducted per PRISMA guidelines. We performed pooled analysis on safety outcomes from six studies which administered FOLFOX (5-fluorouracil, leucovorin, and oxaliplatin) or FOLFIRI (5-fluorouracil, leucovorin, irinotecan) as backbone chemotherapy. Thirty different adverse events from six categories were compared. First-line bevacizumab-based chemotherapy substantially lowered the risks of adverse events related to the dermatological (RR 0.24, 95% CI: 0.11–0.53, *p* < 0.00001) and renal systems (RR 0.57, 95% CI: 0.37–0.86, *p* = 0.007), while significantly increasing the incidence of cardiovascular adverse events (RR 4.65, 95% CI: 1.83–11.78, *p* = 0.001). Thus, first-line cetuximab-based chemotherapy increases patient susceptibility to dermatological and renal adverse events, especially with rash and electrolyte disorders, whereas bevacizumab-based chemotherapy increases cardiovascular risks such as hypertension and arrhythmia.

## 1. Introduction

Colorectal cancer (CRC) is the third most common cause of cancer-related mortality worldwide, accounting for more than 850,000 deaths per year [1]. The average 5-year survival rate in CRC patients is 63%; however, the 5-year survival rate is substantially lower, less than 20%, in patients initially diagnosed with metastatic colorectal cancer (mCRC), which accounts for 20% of new CRC diagnoses [1]. Moreover, about 25% of patients diagnosed with localized CRC are at elevated risk for metastatic progression in the later course of the disease. In Korea, approximately 15–25% of the patients show synchronous metastases and 30–40% of them have unresectable liver metastasis [2]. Ultimately, 50–60% of patients present with mCRC, including recurrent metachronous CRC [3]. The treatment modalities for mCRC include surgical resection and medication management, involving chemotherapy, targeted therapy, and immunotherapy; however, mCRC is often unresectable, especially with high cancer stage or the involvement of major organs and remote lymph nodes [3].

For mCRC patients without rat sarcoma viral oncogene homolog (RAS) mutation, chemotherapy with targeted therapeutic agent is usually reserved as first-line therapy for unresectable mCRC patients [3]. The current National Comprehensive Cancer Network (NCCN) guideline recommends two different classes of targeted therapeutic agents with backbone chemotherapy including FOLFOX (5-fluorouracil, leucovorin, and oxaliplatin), FOLFIRI (5-fluorouracil, leucovorin, and irinotecan), CAPEOX (capecitabine and oxaliplatin) and FOLFOXIRI (5-fluorauracil, leucovorin, oxaliplatin, and irinotecan) in mCRC patients: vascular endothelial growth factor (VEGF) and epidermal growth factor receptor (EGFR) inhibitors. Bevacizumab, a recombinant humanized VEGF antibody, inhibits tumor angiogenesis, whereas EGFR inhibitors, such as cetuximab and panitumumab, block tumor cell proliferation [3,4]. Currently, bevacizumab is the most commonly prescribed first-line targeted therapeutic agent in patients with unresectable synchronous mCRC as part of conversion therapy [3]. EGFR inhibitors, on the other hand, are only recommended in patients with RAS wild-type left-sided mCRC, as EGFR antibodies only elicit responses in patients with RAS wild-type mCRC, implying that genetic variability is significantly associated with patient outcomes [3]. Previous studies demonstrated superior efficacy of cetuximab over bevacizumab [4,5]; however, comprehensive comparative analysis on tolerability, especially on serious adverse events (SAEs) in patients receiving cetuximab or bevacizumab is still lacking despite markedly increased the risk of adverse events (AEs) with concomitant administration of chemotherapy such as 5-fluorouracil, leucovorin, irinotecan, or oxaliplatin [6]. In general, the choice of first-line treatment should be influenced by the tolerability of the medication as well as the patient’s characteristic and efficacy outcomes [6]. Thus, the purpose of the study is to assess tolerability, in terms of comprehensive systemic SAE profiles, of bevacizumab and cetuximab, and to provide guidance on the selection of appropriate targeted therapeutic agents in patients with RAS wild-type tumors.

## 2. Materials and Methods

### 2.1. Data Sources and Search Strategy

This study was conducted according to the Preferred Reporting Items for Systematic Reviews and Meta-Analyses (PRISMA) guidelines [7]. We searched MEDLINE (PubMed), the Cochrane Central Register of Controlled Trials (CENTRAL), and ClinicalKey without year and language restrictions. The initial database search involved a combination of keywords and Medical Subject Headings (MeSH): ‘colon cancer,’ ‘colorectal cancer,’ ‘bevacizumab,’ ‘cetuximab,’ and ‘metastatic cancer’ in the title/abstract. The last search was conducted in March 2021. We manually searched the references of eligible review articles to identify additional studies for meta-analysis.

### 2.2. Study Selection and Data Extraction

Two reviewers independently screened the titles and abstracts of all studies identified in the initial database searches to verify eligibility. Disagreements on study eligibility were further discussed, and any disagreements not meeting consensus were resolved by the third researcher. We extracted the following information from eligible studies: study characteristics (name of the first author, year of publication, study periods, study design, clinical study phase, if applicable, and study region), study population (inclusion criteria for the study and number of patients assigned to each treatment arm), study interventions and comparators (medication names, dosages, types of backbone chemotherapy), and safety outcomes. The safety outcomes included any AEs classified as grades 3 (severe AEs) or 4 (life-threatening or disabling AEs) per common terminology criteria for Adverse Events (CTCAE) version 5.0 [8], and we classified each AE according to the affected physiological systems, such as hematological, dermatological, gastrointestinal (GI), neurological, cardiovascular (CV), and renal systems. Duplicated studies, commentaries, editorials, case reports, clinical trial protocols, review articles, and studies written in languages other than English were excluded. Additionally, any study for which the full text was unavailable was excluded from the analysis. The PICOS (patient, intervention, comparator, outcomes, study design) summary is described in Table 1.

### 2.3. Assessment of Bias Risk and Evidence

Two independent reviewers evaluated the methodological quality of randomized controlled trials (RCTs) utilizing the Cochrane risk-of-bias (RoB) tool [9]. The studies were graded as low, unclear, or high in the following domains: selection bias (random sequence generation and allocation concealment), performance bias (blinding of participants and personnel), detection bias (blinding of outcome assessment), attrition bias (incomplete outcome data), reporting bias (selective reporting), and other potential biases. The observational studies were assessed with the Risk Of Bias in Non-Randomized Studies (ROBINS-I) assessment tool [10], and scored as low, unclear, and high in the following domains: selection of participants, confounding variables, measurement interventions, blinding for assessment, incomplete outcome data, and selective outcome reporting. Disagreements about the risk of bias and quality of evidence were resolved by consensus, and any disagreements not meeting the consensus were resolved by a third reviewer. Funnel plots were used to detect possible publication bias; symmetric funnel plots implied a low risk of publication bias.

### 2.4. Statistical Methods

The risks of AEs were analyzed with relative risks (RRs), and 95% confidence intervals (CIs) were calculated to estimate the risk in mCRC patients undergoing targeted therapies. Reverse percentages were calculated for any results reported as percentages (%) in the original studies. Heterogeneity across the eligible studies was assessed by Cochran’s Q test (significance was revealed for *p* < 0.10) and the I^2^ index [11]. Mantel–Haenszel’s random-effects model was adapted for study outcomes with I^2^ > 50%, which is considered highly heterogenous, whereas a fixed-effects model was used for study outcomes with low heterogeneity (I^2^ < 50%). P values were calculated by two-sided tests, and *p* < 0.05 was considered statistically significant. We utilized Review Manager (RevMan) 5.4 (The Cochrane Collaboration, 2020) for all statistical analyses.

## 3. Results

### 3.1. Study Search and Selection

**Figure 1:**
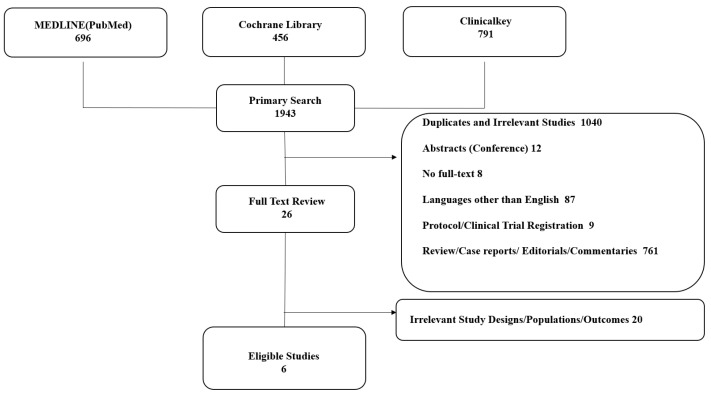
PRISMA Plot.

The primary literature search of MEDLINE (PubMed), the Cochrane Library, and ClinicalKey yielded 1943 studies (Figure 1). A total of 26 of these studies were eligible for full-text reviews after excluding duplicates or irrelevant studies, including those with irrelevant study designs (1040), abstracts including conference abstracts (12), study protocols or clinical trial registrations (9), reviews, case reports, editorials, or commentaries (761), studies without full-text availability (8), and studies written in languages other than English (87). A total of 6 studies [12,13,14,15,16,17] were eligible for the analysis after excluding studies with different study designs and outcomes (20). Therefore, this study included 2498 patients diagnosed with mCRC; a total of 1308 patients received bevacizumab-based chemotherapy, whereas 1190 patients received cetuximab-based chemotherapy as first-line treatments

### 3.2. Study Characteristics

The characteristics of the included studies are described in Table 2. This analysis included three RCTs [12,13,14] and three observational studies [15,16,17]. All studies included RAS wild-type Stage IV or mCRC patients who received bevacizumab- or cetuximab-based chemotherapy as first-line treatments. The majority of study populations received either FOLFOX (mFOLFOX6; 5-fluorouracil, leucovorin, and oxaliplatin) [12,13,15,16,17] or FOLFIRI (5-fluorouracil, leucovorin, and irinotecan) [13,14,15,16,17] as backbone chemotherapy. The study regions included Austria [14], Canada [13], China [15,17], Germany [14], Japan [12], Turkey [16], and the United States [13]. The baseline characteristics of the study populations, such as age, gender, and Eastern Cooperative Oncology Group (ECOG) status, were well balanced between the arms in the original studies. The intervention AE outcomes were classified into the hematological (neutropenia, febrile neutropenia, anemia, thrombocytopenia, hematological toxicity, thrombosis/thromboembolism, infection and hemorrhage), dermatological (mucositis, rash, paronychia, hand-foot syndrome (HFS), GI (nausea, vomiting, diarrhea, GI perforation, liver toxicity, constipation, ileus, and subileus), neurological (peripheral neuropathy and fatigue), CV (hypertension and arrythmia), and renal (nephrotoxicity, proteinuria, dehydration, edema, and electrolyte disorders) systems. Quality assessments of the included studies are reported in Supplementary Tables S1 and S2. The risk of bias was generally acceptable, as implied by the symmetric funnel plots (Supplementary Figure S1).

### 3.3. Safety Outcomes

**Figure 2:**
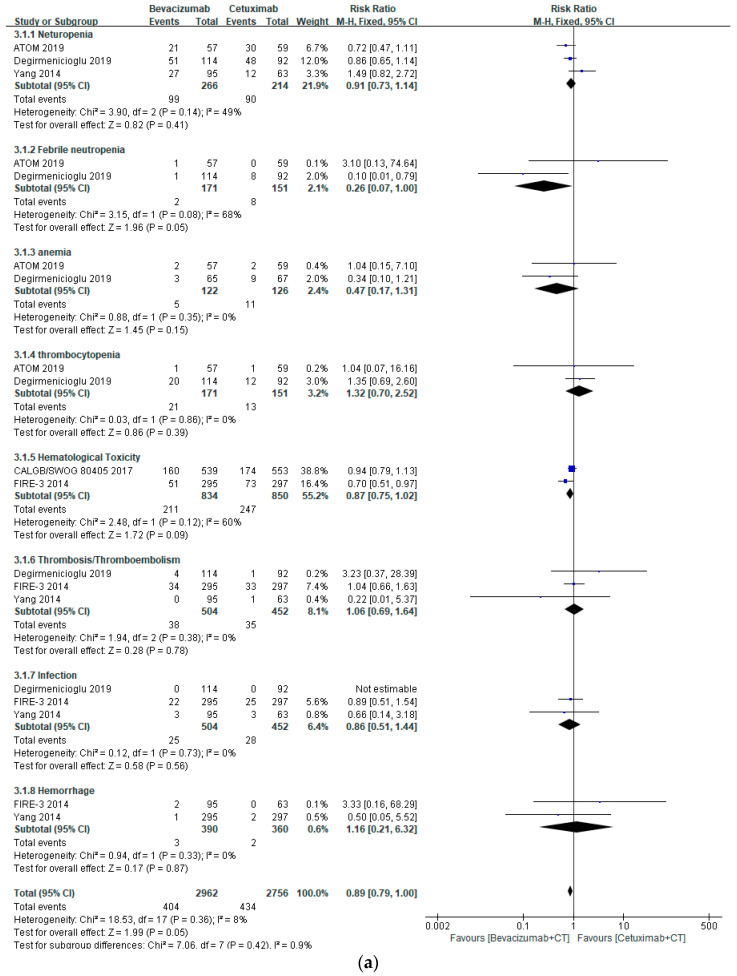

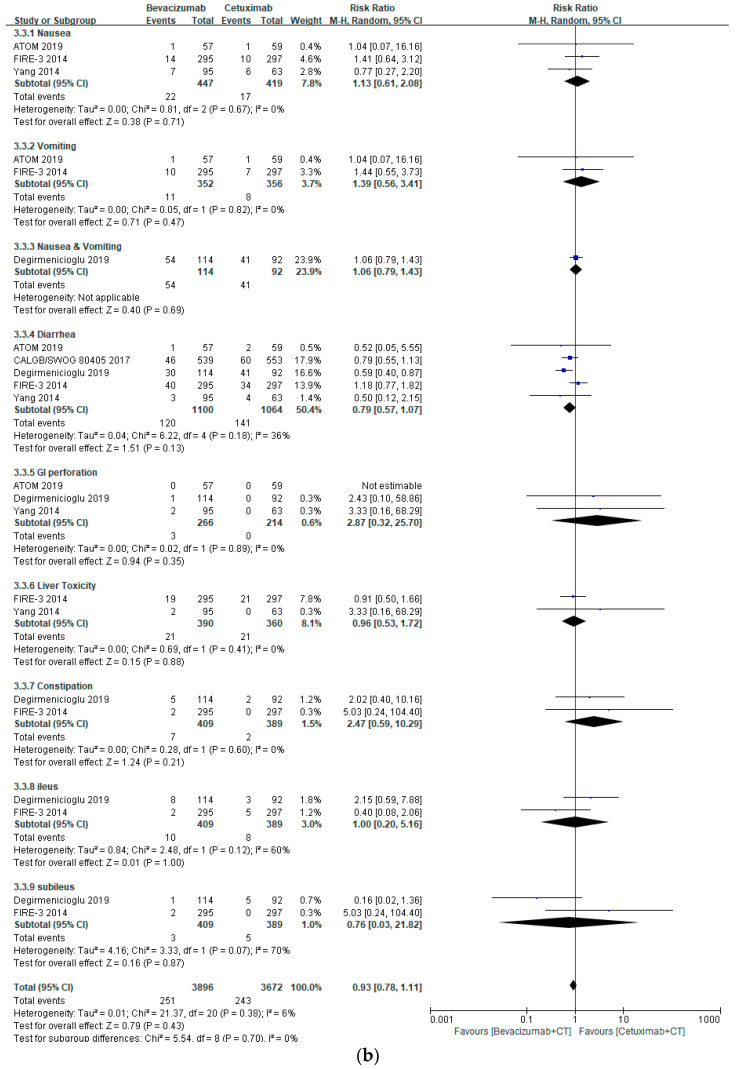

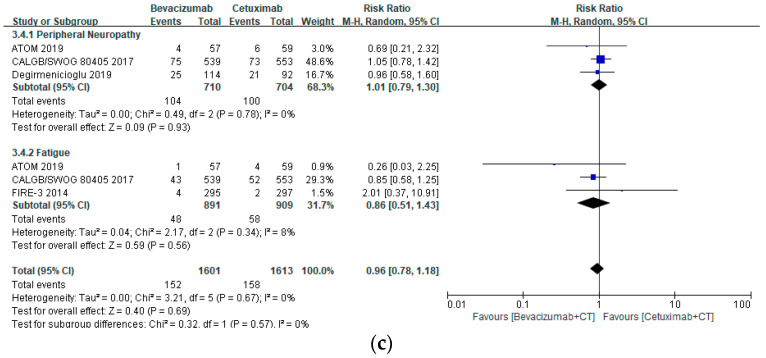
Forest plots of SAEs associated with bevacizumab- and cetuximab-based chemotherapy: (**a**) hematological SAEs, (**b**) GI SAEs, and (**c**) neurological SAEs.

**Figure 3:**
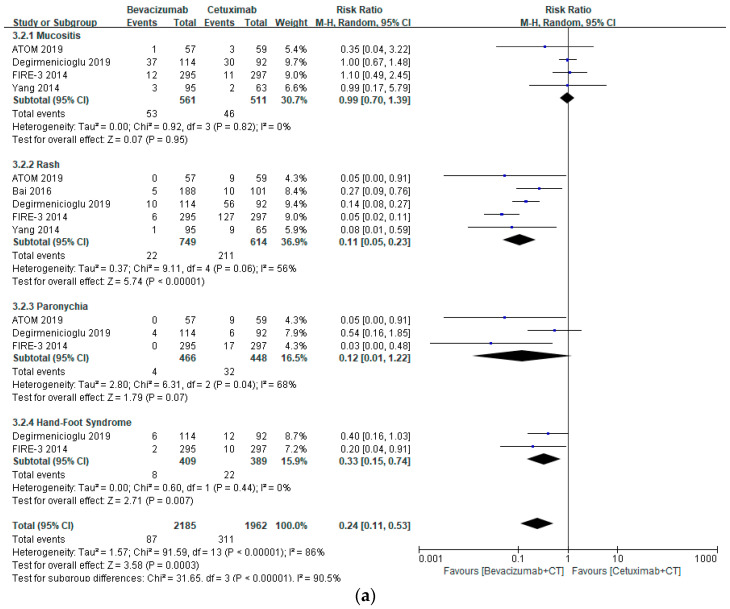

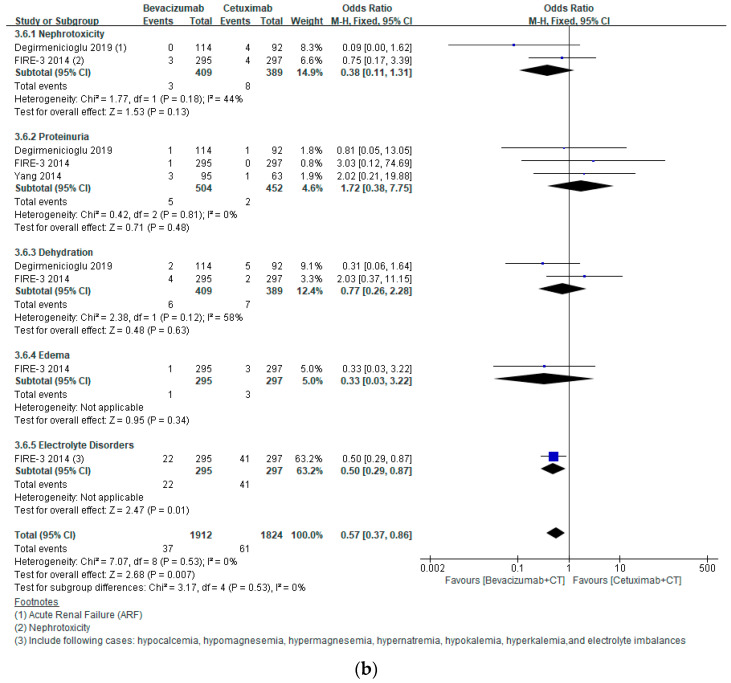
Forest plots of dermatological and renal SAEs associated with bevacizumab- and cetuximab-based chemotherapy: (**a**) dermatological SAEs, and (**b**) renal SAEs.

**Figure 4:**
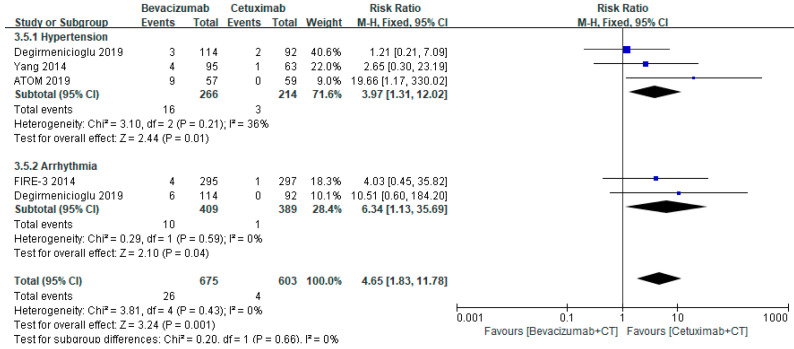
Forest plot of CV SAEs associated with bevacizumab- and cetuximab-based chemotherapy.

The risks of hematological (RR 0.89, 95% CI 0.79–1.00, *p* = 0.05), GI (RR 0.93, 95% CI 0.78–1.11, *p* = 0.42), and neurological AEs (RR 0.96, 95% CI 0.78–1.18, *p* = 0.69) were similar between bevacizumab- and cetuximab-treated patients (Figure 2a–c). However, bevacizumab-based chemotherapy has lower risks of dermatological (RR 0.24, 95% CI 0.11–0.53, *p* = 0.0003) and renal AEs (RR 0.57, 95% CI 0.37–0.85, *p* = 0.007), especially rash (RR 0.11, 95% CI 0.025–0.23, *p* < 0.00001), HFS (RR 0.33, 95% CI 0.15–0.74, *p* = 0.007), and electrolyte disorders (RR 0.50, 95% CI 0.29–0.87, *p* = 0.007) (Figure 3a,b). However, the risk of CV SAEs, including hypertension and arrhythmia, was markedly elevated in patients treated with bevacizumab (RR 4.65, 95% CI 1.83–11.78, *p* = 0.001) (Figure 4).

## 4. Discussion

Combination chemotherapy with cetuximab or bevacizumab in conjunction with the classic CRC treatment regimen has ultimately led to a decreased rate of tumor progression and a significant increase in favorable prognostics of mCRC [3]. Nonetheless, the risk of AEs is markedly attenuated with targeted therapy administered concomitantly with backbone chemotherapy (FOLFOX, FOLFIRI, CAPEOX, or FOLFOXIRI) for CRC [18,19]. According to a retrospective study, the risk of AEs is substantially elevated with drug combinations and intravenous chemotherapy administration, being the major route of administration of both chemotherapy and targeted therapy agents in mCRC patients [6]. However, up to now, a choice of effective first-line biologic chemo-treatment with either cetuximab or bevacizumab in patients with RAS wild-type for mCRC is a still controversial. A recent meta-analysis was performed to determine the efficacy of first-line cetuximab- versus bevacizumab-based chemotherapy for RAS wild-type mCRC [5]. According to these results, it seems reasonable to initiate treatment of RAS wild-type mCRC patients with an anti-EGFR strategy [5]. However, an equally important aspect for achieving complete clinical success is the prevention of avoidable AEs. They only compared the toxicity burden of both hematologic adverse events and nonhematologic adverse events, which were comparable between the two groups [5]. However, considering that chemotherapy induces numerous AEs into the whole body system, classifying AEs in to two categories, hematologic and nonhematologic AEs, is not sufficient to predict patient outcomes. Furthermore, the SAE risks from bevacizumab- or cetuximab-mased chemotherapy should be evaluated to improve patient outcomes by preventing avoidable SAEs.

In our study, we comprehensively investigated 30 different systemic SAE (Grade 3 and 4 per CTCAE) profiles of bevacizumab- and cetuximab-based chemotherapy to assess the tolerability of targeted therapy in treatment-naïve patients diagnosed with RAS wild-type mCRC. Our pooled analysis revealed no differences in the risks of hematological, GI, and neurological SAEs between bevacizumab- and cetuximab-based chemotherapies; however, bevacizumab-based chemotherapy has lower risks of dermatological (RR 0.24, 95% CI 0.11–0.53, *p* = 0.0003) and renal SAEs (RR 0.57, 95% CI 0.37–0.86, *p* = 0.007) than cetuximab-based chemotherapy.

A review article evaluating cetuximab AEs from 15 trials with cetuximab monotherapy and combination cetuximab therapy suggested that more than 80% of patients receiving cetuximab reported dermatological AEs including papulopustular or acneiform skin rash and nail disorders [18]. On the other hand, the PRODIGE18 (Partenariat de Recherche en Oncologie DIGEstive) randomized clinical trial compared the AEs between cetuximab-based and bevacizumab-based chemotherapies in 132 patients with mCRC. They showed comparable dermatologic AEs with any and grade 3/4 (skin disorders, stomatitis, and paronychia) with no significant differences [20]. Similarly, our systematic meta-analysis also revealed insignificant elevation in the risk of mucositis and paronychia in patients treated with cetuximab when compared with bevacizumab, but the risk of rash and HFS was markedly lower in patients receiving bevacizumab-based chemotherapy with RRs of 0.11 (95% CI 0.05–0.23, *p* < 0.00001) and 0.33 (95% CI 0.15–0.74, *p* = 0.007) for rash and HFS, respectively. According to a meta-analysis of the cetuximab-induced rash, the risk of cetuximab-induced high grade acneiform rash was substantially elevated when administered with concomitant chemotherapy, indicating that mCRC patients are vulnerable to papulopustular or acneiform skin rash [21], and a cohort demonstrated a greater risk of cetuximab-induced rash in male and young patients [22]. Nonetheless, cetuximab administration should not be interrupted as cetuximab-induced grade 2–4 dermatological toxicity is dose-dependent and has a strong correlation with progression-free survival (PFS), and overall survival (OS) prolongation in mCRC patients though pharmacological management is currently limited to moisturizing skin products for dermatological disorders [23].

Electrolyte disorders, such as hypomagnesemia, hypokalemia, and hypocalcemia, are commonly reported SAEs related to renal system in patients undergoing cetuximab-based chemotherapy. Previous studies have identified old age, high baseline magnesium levels, and longer treatment duration (usually more than 3 months) as risk factors for cetuximab-induced grade 3 or 4 hypomagnesemia with an incidence of 6–47%, which is mostly reversible after treatment discontinuation; however, delayed management may increase mortality associated with fatal events, including cardiac arrhythmia [23,24]. Despite its substantial impact on patient prognosis, the pharmacological management is limited to electrolyte supplementation for electrolyte disruptions [23]. Hence, more studies on risk stratification as well as SAE management modalities are warranted to improve patient outcomes from cetuximab-based chemotherapy.

On the other hand, bevacizumab has shown substantially elevated SAE risks related to the CV system, including hypertension and arrythmia (RR 4.65, 95% CI 1.83–11.78, *p* = 0.001). Hypertension is one of the most common bevacizumab-induced AEs affecting 4–35% of patients [25]. The mechanism of bevacizumab-induced hypertension is still being debated, but reduced production of nitric oxide in the endothelium from VEGF inhibition seems to play a crucial role in increasing blood pressure. The incidence of bevacizumab-induced hypertension is dose-dependent, and paradoxically, some studies suggest hypertension incidence may be a marker for predicting improved survival of CRC patients; nonetheless, uncontrolled bevacizumab-induced hypertension may attenuate the risk of other CV disorders, including arrhythmias, ischemic heart disease, and congestive heart failure [25]. Although current guidelines do not offer specific recommendation for bevacizumab-induced hypertension in patients with underlying CV disorders, stratification of CV risks is required in patients who are planning bevacizumab-based chemotherapy. It can be easily aggregable since bevacizumab is the most commonly prescribed targeted therapeutic agent and patients with underlying CV disorders or the risk factors are at elevated risk of CV-related hospitalization due to bevacizumab treatment [26]. Furthermore, a recent meta-analysis on fatal adverse events (FAE) of targeted therapeutic agents in CRC patients revealed the highest FAE cases associated with CV events despite similar incidence of fatal AEs between bevacizumab and cetuximab [27]. Hence, the development of standard CV monitoring parameters associated with bevacizumab administration should be endorsed.

To the best of our knowledge, this is the first meta-analysis comprehensively analyzing 30 individual SAE profiles of bevacizumab and cetuximab prescribed as first-line therapeutic agents for RAS wild-type mCRC. In the PRODIGE18 trial, an incidence ratio of 15 AEs was assessed among small number of patients of 65 and 67 patients in bevacizumab and cetuximab groups, respectively, and no statistical comparison was performed [20]. Thus, the evaluation of potential risk of AE occurrence between two major targeted therapy-based chemotherapy of RAS wild-type mCRC is beneficial for proper management of the entire clinical profile of the patients. We also assessed hepatic and renal toxicity in overall and the related components between the bevacizumab- and cetuximab-based chemotherapy among patients with RAS wild-type mCRC. Furthermore, the risk of infection, the major complication of chemotherapy secondary to neutropenia [28], was not substantially different between cetuximab (*n* = 452)- and bevacizumab (*n* = 405)-based chemotherapy for RAS wild-type mCRC. Nonetheless, caution is required when interpreting the study results because AEs are induced by numerous factors including older age, multiple comorbidities, drug combination, drug interactions, route of administration, and treatment duration [6]. Although this analysis included two backbone chemotherapy regimens, FOLFOX and FOLFIRI, variable chemotherapy regimens options including CAPEOX and FOLFOXIRI are available for mCRC patients, and a different combination may induce divergent AEs [3]. Moreover, administration of drug combination regimen may predispose patients at escalated risk of AEs secondary to drug–drug interactions [29], and studies suggest that approximately 27–58% of cancer patients experience at least one drug–drug interaction during cancer management [30,31]. However, due to the large burden of medications and the substantial toxicity of chemotherapeutic agents, the causes of AEs during cancer management may obscure, and severely attenuated patients’ health status from metastatic cancers may disguise the AEs [3]. Thus, assumptions that all cancer patients possess increased AE risks associated with chemotherapy regimen, drug–drug interactions and patient status should be taken into consideration during patient care.

Based on the previous studies [4,5], cetuximab-based chemotherapy has higher efficacy as implied by the superior response rate, which consequently increases patient survival; however, further assessment of tolerability of cetuximab-based chemotherapy should not be neglected because of substantially elevated AE risks of electrolyte and dermatological disorders, which may decrease patients’ quality of life (QOL). In fact, a prospective cohort study showed better patient-related outcomes and health-related QOL measured by European Organization for the Research and treatment of Cancer Quality of Life Questionnaire (QLQ)-C30 and QLQ-CR 29 questionnaires in mCRC patients treated with bevacizumab compared with patients receiving cetuximab-based chemotherapy [32]. The clinical outcomes from medication management in metastatic cancer patients are unpredictable in most cases because numerous factors, including patient-specific factors (age, comorbidities, genetics, and health status inferred by ECOG score), cancer characteristics, chemotherapy regimens, and drug–drug interactions can affect patients’ responses to medication, and patients diagnosed with metastatic cancers already possess underlying factors associated with increased AE risk [3,25]. Thus, risk stratification related to SAEs in addition to medication efficacy should be taken into consideration when choosing medication regimens for RAS wild-type mCRC patients.

The interest in precision medicine in cancer has been steadily increased and has advanced the clinical diagnosis and management of cancer [33,34]. The clinical efficacy of chemotherapeutic agents as well as patient prognosis are easily predicted by the precision medicine, and administration of EGFR inhibitors in RAS wild-type mCRC patients is an example of precision medicine, in terms of variable drug responses secondary to genetic traits [33]. Recently, computational methods involving virtual molecular tumor boards, digital twins, and dynamic precision medicine have be adapted for precision oncology trials on cancer biomarkers, thereby improving patient outcomes by selecting optimal therapeutic agents [35]. However, the application of precision medicine on the prediction of chemotherapy-related AEs is relatively neglected despite the significant AE risks in cancer patients, as implied by the limited number of studies. As previously stated, the causes of AEs are variable, subsequently making AE predictions ambiguous. Therefore, further studies on mechanism of AEs of each chemotherapeutic and targeted agents as well as AE traits associated with genetic variability are warranted to establish a foundation on precision medicine-based AE prediction modeling to improve patient outcomes.

This study possesses some limitations that should be acknowledged. First, the included studies had different study designs and outcome measurements. All patients received either FOLFOX or FOLFIRI; thus, the backbone chemotherapy regimens are not the same among the studies. Moreover, only two RCTs investigated our outcomes in mCRC patients administering the same backbone chemotherapeutic regimens; others were observational studies retrospectively recruited patients who received either bevacizumab- or cetuximab-based chemotherapy, which increased the heterogeneity across the study outcomes. Nonetheless, monotherapy is rare in cancer management, and patients receive at least two concomitant chemotherapeutic agents to improve clinical outcomes, which subsequently makes patients more susceptible to unpredictable AEs. However, since metastatic cancer patients are vulnerable population who rarely participate in clinical trials and for whom various regimens are available, this study has strong external validity because we comprehensively evaluated the overall AE profiles of targeted therapy administered by diverse chemotherapy regimens. However, further research on factors associated with risk stratifications of AEs is recommended to improve the clinical outcomes of mCRC patients

## 5. Conclusions

First-line cetuximab-based chemotherapy may predispose treatment-naïve mCRC patients at elevated risk for dermatological and renal SAEs, mostly rash, HFS, and electrolyte disorders. On the other hands, mCRC patients undergoing first-line bevacizumab-based chemotherapy may have higher CV risks such as hypertension and arrhythmia. However, the risks of hematological, GI, and neurological SAEs are comparable between cetuximab- and bevacizumab-based chemotherapy patients.

## Figures and Tables

**Table 1:** PICOS of the study.

Component	Definition
**P (patients)**	Patients diagnosed with RAS wild-type metastatic CRC who were administered the intervention or the comparator as first-line treatments
**I (intervention)**	Bevacizumab + chemotherapy
**C (comparator)**	Cetuximab + chemotherapy
**O (outcomes)**	SAEs (GRADE 3–4): **Hematological SAEs:** neutropenia, febrile neutropenia, anemia, thrombocytopenia, hematological toxicity, thrombosis/thromboembolism, infection, and hemorrhage **Dermatological SAEs:** mucositis, rash, paronychia, and HFS GI AEs: nausea, vomiting, diarrhea, GI perforation, liver toxicity, constipation, ileus, and subileus **CV SAEs:** hypertension and arrhythmia **Neurological SAEs:** peripheral neuropathy and fatigue **Renal SAEs:** proteinuria, dehydration, edema, and electrolyte disorders
**S (study design)**	RCTs and observational studies

Abbreviations: CRC, colorectal cancer; CV, cardiovascular; GI, gastrointestinal; HFS, hand-foot syndrome; RCT, randomized controlled trials; SAE, serious adverse events.

**Table 2:** Characteristics of included studies.

Study Name	Study Duration	Country	Study Design	Patient Population	Intervention	Comparator	Backbone CT	Outcomes
RCTs								
ATOM (Oki et al.) 2019 [12]	May 2013–April 2016	Japan	Multicenter, randomized phase II study	Patients aged between 20 and 80 years with liver-limited metastases from wt (K) RAS CRC	Bevacizumab (5 mg/kg) (*n* = 57)	Cetuximab (400 mg/m^2^ first dose followed by 250 mg/m^2^ on Day 1 through Day 2) (*n* = 59)	mFOLFOX6	Hematological, dermatological, GI, neurological, and CV AEs
CALGB/SWOG 80405 (Venook et al. 2017) [13]	November 2005–March 2012	United States and Canada	Multicenter, randomized phase III study	Patients aged ≥18 years with previously untreated advanced or metastatic colorectal cancer whose tumors were KRAS wt	Bevacizumab (5 mg/kg) (*n* = 559)	Cetuximab (400 mg/m^2^ followed by 250 mg/m^2^ weekly) (*n* = 578)	mFOLFOX6 or FOLFIRI	Hematological, GI, and neurological AEs
FIRE-3 (Heinemann et al. 2014) [14]	23 January 2007–19 September 2012	Germany, Austria	Randomized, open-label phase 3 trial	Patients aged 18–75 years with stage IV, histologically confirmed, adenocarcinoma of the colon or rectum, ECOG performance status of 0–2, an estimated life expectancy of greater than 3 months and adequate organ function, and no surgery within the 4 weeks before the study	Bevacizumab (5 mg/kg) (*n* = 295)	Cetuximab (400 mg/m^2^ on Day 1 and 250 mg/m^2^ weekly) (*n* = 297)	FOLFIRI	Hematological, dermatological, GI, neurological, CV, and renal AEs
Observational								
Bai et al. 2016 [15]	January 2009–December 2013	China	Observational cohort study	Patients with histologically proved stage IV CRC who have consecutively received at least 2 courses of bevacizumab-based (KRAS wt or mutated) or cetuximab-based (KRAS wt) triplet biochemotherapy as their first line	Bevacizumab (5 mg/kg biweekly or 7.5 mg/kg triweekly) (*n* = 188)	Cetuximab (400 mg/m^2^ first dose, 500 mg/m^2^ biweekly or 750 mg/m^2^ triweekly) (*n* = 101)	mFOLFOX-6, FOLFIRI	Dermatological AEs
Degirmenicioglu et al. 2019 [16]	Not specified	Turkey	Retrospective multicenter observational study	Patients diagnosed pathologically as CRC adenocarcinoma with KRAS wt and who received chemotherapy in combination with either bevacizumab, cetuximab or panitumumab	Bevacizumab (*n* = 114)	Cetuximab (*n* = 92)	FOLFOX, FOLFIRI	Hematological, dermatological, GI, neurological, CV, and renal AEs
Yang et al. 2014 [17]	April 2005–March 2012	China (Taiwan)	Retrospective cohort	Patients with histologically proven colorectal cancer at clinical stage IVa or IVb according to AJCC VII and who received at least 4 courses of bevacizumab-based or cetuximab-based triplet biochemotherapy as first-line treatments	Bevacizumab (5 mg/kg biweekly) (*n* = 95)	Cetuximab (500 mg/m^2^ biweekly) (*n* = 63)	FOLFIRI, FOLFOX	Hematological, dermatological, GI and CV AEs

Abbreviations: AE, adverse events; AJCC, American Joint Committee on Cancer; CR, complete response; CrCl, creatinine clearance; CRC, colorectal cancer; CV, cardiovascular; ECOG, Eastern Cooperative Oncology Group; FOLFIRI, 5-fluorouracil/leucovorin and irinotecan; FOLFOX, 5-fluorouracil/leucovorin and oxaliplatin; GI, gastrointestinal; KRAS, Kirsten Rat Sarcoma; wt, wild-type.

## Data Availability

The data presented in this study are available in article or supplementary material cited in the reference.

## Supplementary Materials

The following supporting information can be downloaded at: https://www.mdpi.com/article/10.3390/healthcare10020217/s1, Figure S1: Funnel plot of hematological adverse events, Table S1: Quality assessment of included RCTs, Table S2: Quality assessment of included observational studies.
