# Supplementary material for: Tolerability on Serious Adverse Events of First-Line Bevacizumab and Cetuximab for RAS Wild-Type Metastatic Colorectal Cancer: A Systematic Review and Meta-Analysis
Source: Healthcare (Basel). 2022 Jan 23;10(2):217. doi: 10.3390/healthcare10020217 (PMC8871808; doi:10.3390/healthcare10020217)

Supplementary Table S1. Quality assessment of included RCTs

RCTs	Random sequence generation	Allocation concealment	Blinding of participants and personnel	Blinding outcome assessment	Incomplete outcome data	Selective reporting	Other bias
ATOM (Oki 2019)	L	L	U	U	L	L	L
CALGB 80405 (Venook 2017)	L	L	H	H	L	L	L
FIRE-3 (Heinmann 2014)	L	L	H	H	L	L	U

Supplementary Table S2. Quality assessment of included observational studies

Observational Studies	Selection of participants	Confounding variables	Measurement of intervention	Blinding for outcome assessment	Incomplete outcome data	Selective outcome reporting
Bai et al -2016	L	H	L	H	L	L
Dreanic et al 2015	L	U	L	H	U	U
Degirmencioglu et al 2019	L	L	L	H	H	H
Kim et al 2020	L	U	L	H	L	L
Marques et al 2020	L	H	L	H	U	U
Sagawa et al 2020	L	U	L	H	L	L
Yang et al 2014	L	U	L	H	L	U

Supplementary Figure S1. Funnel plot of hematological adverse events

a)

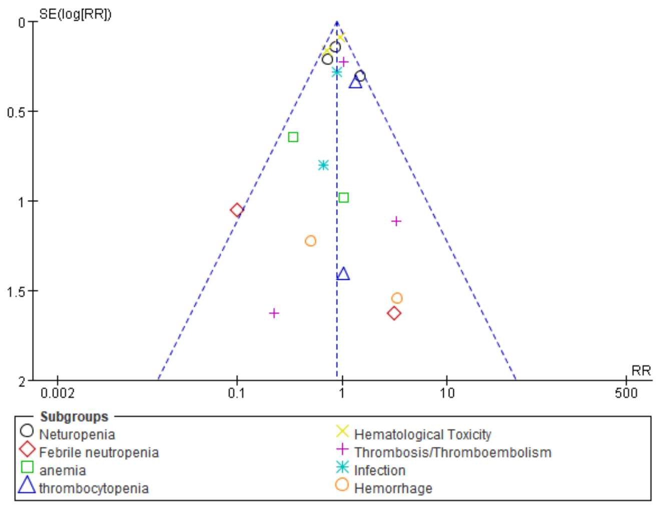

Supplement: Supplementary file 1 [file healthcare-10-00217-s001.zip › healthcare-1533555-supplementary.pdf]
